# Does the Mexican sugar-sweetened beverage tax have a signaling effect? ENSANUT 2016

**DOI:** 10.1371/journal.pone.0199337

**Published:** 2018-08-22

**Authors:** Cristina Álvarez-Sánchez, Isobel Contento, Alejandra Jiménez-Aguilar, Pamela Koch, Heewon Lee Gray, Laura A. Guerra, Juan Rivera-Dommarco, Rebeca Uribe-Carvajal, Teresa Shamah-Levy

**Affiliations:** 1 Program in Nutrition, Department of Health and Behavior Studies, Teachers College Columbia University, New York, NY, United States of America; 2 Mexican National Institute of Public Health (INSP), Cuernavaca, Morelos, Mexico; Cinvestav-Merida, MEXICO

## Abstract

**Objective:**

To evaluate the potential signaling effect of the Mexican tax on sugar-sweetened beverages (SSBs) by analyzing the association between awareness of and opinions about its effectiveness with current consumption of taxed SSBs and with a self-reported change in consumption of SSBs since the implementation of the tax. We also examined the association between psychosocial and environmental determinants of SSB consumption with current consumption of taxed SSBs and with a reported change in consumption of SSBs.

**Methods:**

Cross-sectional analyses of survey and food-frequency questionnaire data from the Mexican National Health and Nutrition Survey 2016. Participants were Mexican adults (20–59 years, N = 6,650). Logistic regression was used to evaluate the probability of a person reporting a decrease in SSB consumption, given their awareness of the tax, opinion about its effectiveness, psychosocial (SSB health-related beliefs, self-efficacy, and liking of SSBs) and environmental (availability of potable water) determinants. Multiple linear regression analysis was utilized to examine the association between the aforementioned factors and current consumption of taxed SSBs.

**Results:**

Compared with adults not aware, adults who were aware of the SSB tax were more likely (*OR* = 1.30) to report a decrease in SSB consumption (*p* = .012). In urban areas, adults aware of the tax drank a significantly lower amount of taxed SSBs (-15.7%; *p* = .023) than those not aware. Self-efficacy and liking of SSBs were significantly associated with a reported decrease in consumption and with current consumption (*p* < .001), while health beliefs and availability of potable water were not significantly associated with either reported change in SSB consumption or current consumption of taxed SSBs.

**Conclusions:**

Implementation of an SSB tax accompanied by highly visible campaigns may further influence the impact of taxes on SSBs consumption. Future public health and nutrition education campaigns designed to increase knowledge and enhance motivation should be complemented by programs to assist individuals develop self-efficacy and self-regulation skills.

## Introduction

In Mexico, 73% of adults and 36% of children and adolescents (aged 2–19 years) have overweight or obesity [1]. Nearly 15% of adults are estimated to have type 2 diabetes—being the principal cause of mortality [2]. Frequent consumption of SSBs has been linked to an increased risk of a number of adverse health outcomes, including obesity [3–5], type 2 diabetes [6–9], coronary heart disease [10], dental caries [11], and tooth loss [12].

Consumption of SSBs, soda in particular, in Mexico is widespread [13] and has a strong cultural component [14]. Among many groups, soda drinking is a sign of conviviality, hospitality, and even of social status [14]. Currently, SSBs contribute about 69% of added sugars, 45% of total sugar intake, and 10% of total energy intake to the Mexican diet [13], more than three times the level recommended by the American Heart Association and approximately 3% of total energy intake [15, 16].

Due to this context, public health professionals advocated for the passage of an excise SSB tax and carried out strong and focused public awareness campaigns about the sugar content in SSBs, the health consequences of a high SSB consumption, and the rationale of a SSB tax; they also proposed that the SSB tax revenue be used to pay for purified water fountains in schools [17]. The debate around the Mexican SSB tax attracted a considerable amount of media attention and raised the profile of these issues among the public [17]. This culminated in the passing of a nationwide one-peso per liter (equivalent to a 10% increase) excise tax on SSBs [18], levied on manufacturers and effective from January 1, 2014 –along with the implementation of other public health actions–as a public health measure to counteract obesity.

Studies conducted since the implementation of the tax indicate that SSB purchases by Mexican households declined by 7.6% on average in 2014 and 2015, even more than trends predicted [19, 20]. The decrease in purchases suggests a corresponding reduction in SSB consumption and therefore of caloric intake. The decrease in purchases and consumption may not be fully explained by the (economic) elastic nature of SSBs [19], but may be the result of an increased awareness of the detrimental health effects of SSBs. One study conducted prior to the implementation of the tax had already found declines in sales of SSBs in Mexico which, the authors hypothesize may have been due to “[a very] visible and well-funded media campaign linking [SSBs] with diabetes” [21].

Behavioral economics research suggests that the way in which taxes are presented or framed matters and could influence their impact [22]. SSB taxes are believed to provide consumers a behavioral rationale for changes (like nudges), in addition to traditional economic justification [23]. According to Adbukadirov [23], SSB taxes can increase the prominence of beverage choice to consumers through two mechanisms, first, “[SSB taxes] and the publicity that surround[s] [them may] trigger consumers to think about their health goals and to choose healthier drink[s],” and second, “attaching higher costs to unhealthy choices at the time of purchase may help undercut consumers’ myopia by countering the immediate benefits of enjoying a [SSB] with the immediate costs of the [SSB] tax.” There is emerging evidence supporting the hypothesis that “junk food” and SSB taxes imposed with public health goals in mind may contribute to enhancing people’s awareness about the negative health consequences of highly processed, less healthy foods and beverages [24, 25]. In economic theory, this is known as the “signaling effect” of tax policy, which poses that in addition to the tasks of raising public funds and correcting external effects, tax policies signal missing information to individuals about the effect of their consumption of the taxed product [26].

Understanding the impact of the Mexican SSB tax is further complicated by the fact that there were other initiatives undertaken during the same period, including the regulation of unhealthy food and beverages in schools [27, 28], the partial voluntary self-regulation of foods and beverages advertising directed at children [29], and the regulation of advertisement of foods and non-alcoholic beverages during children’s television viewing time [30] that may have had an impact on SSB purchases over the same time period.

While it would be very difficult to evaluate the separate effects of the SSB tax and other simultaneous public health initiatives aimed at curbing SSB consumption, it is important to explore whether awareness of the SSB tax and opinion about its potential to reduce SSB intake, as well as psychosocial and environmental determinants of SSB consumption, are associated with current consumption of taxed SSBs, and with self-reported changes in consumption of SSBs since the SSB tax was passed. To our knowledge, no study has examined these associations after the implementation of a nation-wide SSB tax. Therefore, the current study addressed the following research questions:

1. Are Mexican adults aware of the SSB tax? What is their opinion about the effectiveness of the SSB tax in decreasing purchases of taxed SSB? Do awareness of and opinion about the SSB tax differ by socio-demographic characteristics?
2. Are awareness of and opinion about the effectiveness of the SSB tax, and psychosocial and environmental factors of SSB consumption, associated with a reported decrease in SSB consumption?
3. Are awareness and opinion about the effectiveness of the SSB tax and psychosocial and environmental factors of SSB consumption associated with current consumption of taxed SSBs?

Overall, we hypothesized that a higher percentage of adults living in Mexico City and of higher socio-economic status (SES) would be aware of the tax, and that those who were aware and expressed a positive opinion about the effectiveness of the SSB tax in reducing purchases of SSBs would, in effect, drink less SSBs and/or report a decrease in SSB consumption, compared to those who were not aware and/or expressed a negative opinion about the effectiveness of the SSB tax. These findings would be useful for health advocates and policy makers when considering passing a SSB tax.

## Materials and methods

### Population and study design

The current study is an analysis of data collected with the 2016 Mexican National Health and Nutrition Survey (ENSANUT by its Spanish acronym). The ENSANUT is a nationally representative probabilistic multistage stratified cluster survey constructed with sufficient sampling power to make distinctions between urban (≥ 2,500 inhabitants) and rural (< 2,500 inhabitants) areas, and among four geographic regions (categorized as North, Central, Mexico City, and South). Sampling weights are used to estimate nationally representative values. (A detailed description of the sampling procedures and survey methodology has been described elsewhere [31].) The ENSANUT 2016 was approved by the Research, Ethics and Biosafety Committees at the National Institute of Public Health. Written informed consent was obtained from all study participants. Trained personnel administered all questionnaires and measures face-to-face.

We primarily used data from the *Perception of Obesity*, *Physical Activity and Diet Questionnaire* (POCAA-Q, by its Spanish acronym) [32], which had been applied to a random subsample of 6,550 adults aged 20–59 years. A description about the development and validation of the POCAA-Q can be found elsewhere [33]. The aim of the POCAA-Q was to explore adult Mexicans’ perceptions of their dietary and physical activity habits as well and knowledge about causes and consequences of obesity. The POCAA-Q also explores the population’s awareness of and opinion about the effectiveness of governmental legislation (including the SSB tax) to prevent and control obesity. The specific questions from the POCAA-Q that were used in this study are described below in measures. Additional data were obtained from other ENSANUT 2016 files: the semi-quantitative food frequency questionnaire (SFFQ), and the demographic file (i.e. demographic, socio-economic characteristics and sample weights).

### Measures

#### Awareness of the tax and opinion about the effectiveness of the tax

The variables awareness of the SSB and opinion about the effectiveness of the SSB tax come from the POCAA-Q. Their operational definitions can be found in S1 Table.

#### Self-perception of change in consumption of SSBs

The variable self-perception of change in consumption of SSBs in the two years prior to the survey is a proxy for the time when the SSB tax was implemented. It also comes from the POCAA-Q. Its description can be found in S1 Table.

#### Consumption of taxed SSBs

Beverage consumption was assessed using a SFFQ [34] which was validated for use with Mexican adolescents and adults [35]. The questionnaire includes 140 food items including a variety of sugar-sweetened, unsweetened, or artificially sweetened beverages. To assess consumption of each food item, reported frequency of consumption was converted into grams. To calculate consumption of taxed industrialized SSBs, we summed quantities (g/person/day) of all SSBs subject to the excise tax included in the SFFQ: regular carbonated SSBs, industrialized flavored waters with added sugar, and industrialized fruit nectars with added sugar. Sweetened energy and sports beverages are subject to the SSB tax, but they are not captured by the FFQ; thus they were not contemplated in this study. The data from the SFFQ had already been cleaned and processed following the steps detailed by Ramirez Silva and colleagues [36]; we excluded an additional three individuals with extreme observations (more than 3 SDs the log taxed SSB consumption).

#### Psychosocial and environmental determinants of SSB consumption

The selection of psychosocial and environmental variables from the POCAA-Q was informed by the health literature and includes SSB health-related beliefs [37] (measured with four questions about beliefs on whether SSB consumption contributes to high blood pressure, obesity, diabetes, and dental caries), self-efficacy to decreased consumption of SSBs [38, 39] (measured with one question), degree of liking of SSBs [40] (measured with one question; with a higher score indicating lower preference of SSBs), and availability of free/low-cost water [41] (measured with one question). S1 Table presents the definitions and rationale for choice of each variable.

For SSB health-related beliefs, a composite scale/measure was constructed based on the four health beliefs questions, with one additional point for a “yes” response regarding the belief about each condition. The scale ranged from 0 (reporting ¨no” to all four health beliefs questions) to 4 (reported “yes” to all four questions), with a higher score indicating an incremental agreement with the statements regarding the health damage of SSBs. (Cronbach’s alpha for the scale was 0.844.)

#### Covariates

Socio-demographic variables included were sex (men and women), age (continuous variable), and a validated socio-economic status index [33] (with terciles derived from principal components analysis of eight variables: household building materials; number of bedrooms; basic services infrastructure; ownership of a car, television, radio, and refrigerator). Body mass index (BMI) was calculated as the weight in kilograms divided by the square height in meters (kg/m^2^) [42]. Height and weight were measured using standardized procedures [43, 44]. Values between 10 and 58 kg/m^2^ were considered as valid data [1]. We used the WHO BMI classification: underweight: <18.5, normal weight: 18.5–24.9, overweight: 25.0–29.9, and obesity: ≥ 30.0 [42].

We also included self-reporting of diabetes diagnosis, in response to the question: “¿Algún médico le ha dicho que tiene diabetes o alta el azúcar en la sangre?” (Has a doctor told you that you have diabetes or high blood sugar?).

### Statistical analyses

#### Relationship between categorical variables

Chi-square tests were run to examine the relationship between categorical variables, such as awareness of the SSB tax and socio-demographic variables; with a *p*-value < .05 as the cutoff point for statistical significance. Differences between subcategories of socio-demographic variables (e.g. sex: male/female, location: urban/rural) were considered to be statistically significant if their 95% confidence intervals (CIs) did not overlap; we used this approach recognizing its limitation, namely, that when the CIs of two statistics do not overlap, they are necessarily significantly different, but they could be significantly different even if their CIs overlap [45].

#### Binary logistic regression

A binary logistic regression was conducted to evaluate the probability that a given person would report a decrease in their SSB consumption in the two years prior, given their: (1) awareness of the SSB tax, (2) opinion about the effectiveness of the SSB tax in reducing purchases of SSBs, (3) health beliefs scale (psychosocial determinant), (4) self-efficacy (psychosocial determinant), (5) liking of SSBs, and (6) availability of potable water for free or at a low cost. We constructed the binary outcome variable (consumption of SSBs decreased and consumption did not decrease) from the three-category perception of change in the SSB consumption variable by keeping the “consumption decreased” category and combining the “consumption stayed the same” and “consumption increased” categories. Covariates sex, BMI, SES, geographic region, urban-rural location, and diabetes diagnosis were entered as categorical variables; age was entered as a continuous variable. As indicated by Bursac and colleagues, selection of variables for the logistic regression was conducted purposefully using (manual) backward elimination [46]. We started with a full model with the six variables and seven covariates, tested for interactions, and subsequently eliminated insignificant predictors at four different steps to arrive at a parsimonious final model [46]. The final logistic regression model includes three variables: awareness of the tax, self-efficacy, and liking of SSBs; and two covariates: age and diabetes diagnosis.

Results are expressed as adjusted *Odds Ratio* (*OR*) and their corresponding 95% CIs. Results were considered to be statistically significant if the 95% CI excluded the value of 1 [47].

#### Multiple linear regression

Multiple linear regression analysis was utilized to examine the association between six variables: (1) awareness of the tax, (2) opinion about the effectiveness of the SSB tax, (3) health beliefs scale (psychosocial determinant), (4) liking of SSBs (psychosocial determinant), (5) self-efficacy (psychosocial determinant) and (6) availability of potable water for free or at a low cost; with current consumption of taxed SSBs (log g/d), after controlling for seven covariates (sex, age, BMI, diabetes diagnosis, SES, urban-rural location, and region). The outcome variable (consumption of taxed SSBs (log g/person/day)) was strongly, positively skewed, and thus log-transformed. For the purpose of improving interpretability of the beta estimates, we calculated the percentage change for each estimate in the outcome variable per one unit change in the independent variable while all other variables in the model were held constant; we used the equation: % *change in consumption of taxed SSBs* = (*e^β^* − 1) * 100 [48]. We started with a full model with the six variables and seven covariates, tested for interactions, and subsequently performed a manual backward elimination of insignificant predictors, at three different steps, to arrive at a parsimonious final model where all variables included were significant [47]. The final multiple regression model includes three variables: awareness of the tax, self-efficacy, and liking of SSBs; and the seven covariates: urban-rural location, sex, age, region, diabetes diagnosis, and BMI. Multiple regression results are expressed as: regression coefficients, percent changes in consumption of taxed SSBs in relation to changes in independent variables, and standard errors. The (adjusted) R square is presented to indicate the estimated amount of explained variance. Results were considered significant at *p* < .05 [47].

Lastly, we estimated mean taxed SSB consumption for the total sample and by the theoretical variables of interest (which include awareness and opinion about the tax, as well as psychosocial and environmental factors). All statistical analyses were performed with IBM SPSS, version 24.0. Calculations were weighted by expansion factors and adjusted for the complex sampling survey design using the SPSS command for complex surveys. Data for the χ^2^ tests met the assumptions of sample size and independence of observations. The binary logistic regression model was checked for linearity and multicollinearity. The multiple regression model was checked for multicollinearity, linearity and for normality, homoscedasticity, and independence of residuals.

## Results

Study population characteristics are presented in Table 1.

**Table 1:** Socio-demographic characteristics.

		Un-weighted *n*	Weighted *n* (in millions)	Weighted percentages (%)
Sex				
	Male	2,152	25.7	47.8
	Female	4,498	28.5	52.2
SES				
	Low	2,276	12.4	20.8
	Medium	2,266	17.3	29.1
	High	2,108	29.8	50.1
Location				
	Urban	3,323	46.0	77.3
	Rural	3,327	13.5	22.7
Region				
	North	1,434	12.5	21.0
	Center	2,171	19.6	33.0
	Mexico City	763	10.4	17.6
	South	2,282	17.0	28.4
Age (*mean* ± *SEM*)		38.6 ± 0.1	36.6 ± 0.3	NA
	20–29	1,647	19.1	32.1
	30–39	1,936	17.2	28.9
	40–49	1,694	13.2	22.2
	50–59	1,373	10.0	16.8
BMI (*mean* ± *SEM*)		28.7 ± 0.1	28.5 ± 0.1	NA
	Normal weight	1,582	14.6	26.3
	Overweight	2,423	21.9	39.5
	Obesity	2,316	19.0	34.2
Total		6,650	59.5	100

Notes.

SES, socio-economic status; SEM, standard error of the mean; BMI, body mass index; NA; non-applicable.

Data are from the ENSANUT 2016: Mexican adults (20–59 years old), *n* = 6,650.

### Awareness and opinion about the SSB tax

**Fig 1:**
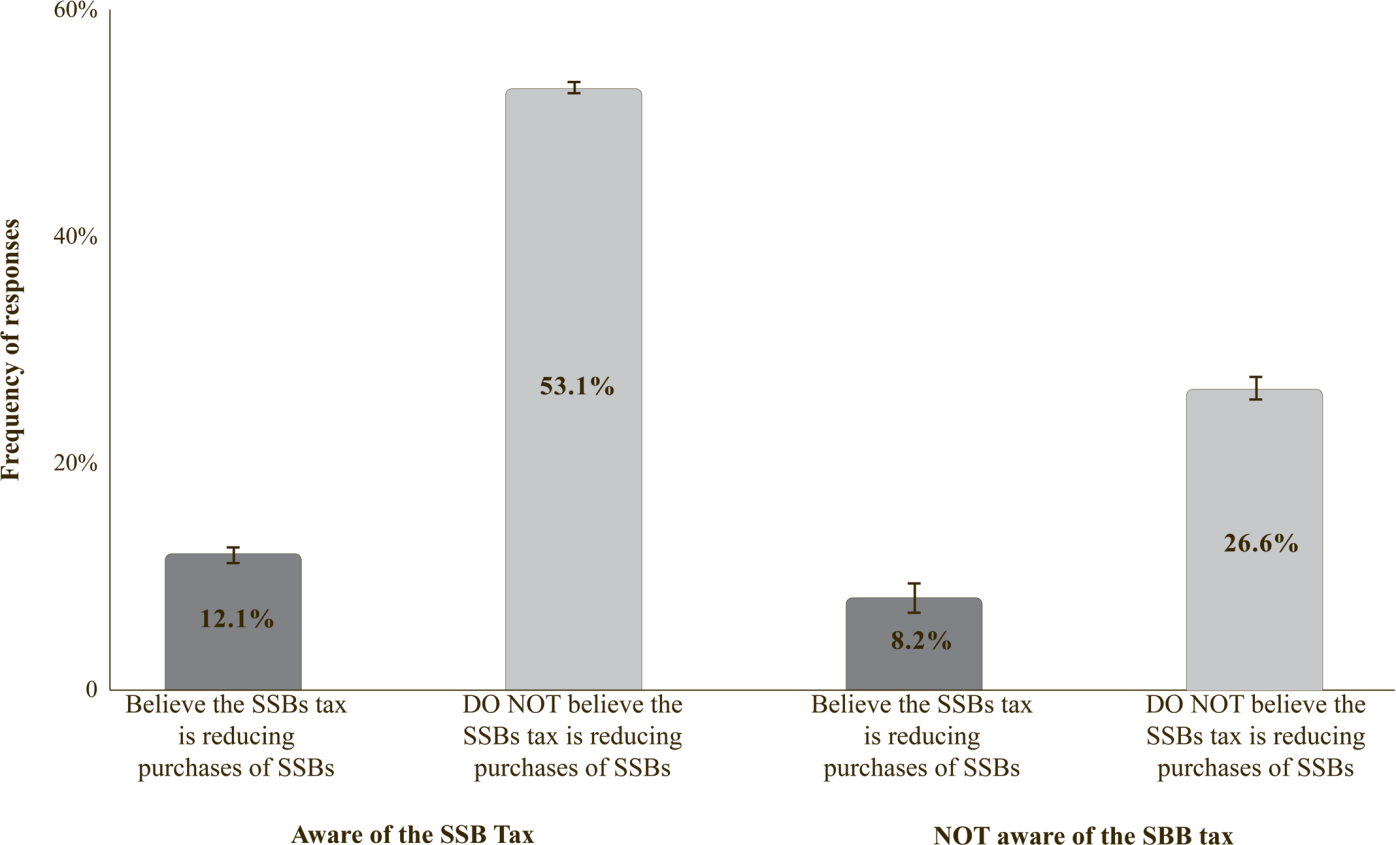
Awareness of the SSB tax and opinion about whether it was reducing the purchases of SSBs (n = 6,321).

At national level, 65.2% of the respondents reported being aware of the existence of the SSB tax, however, only 20.3% indicated that they thought the SSB tax was helping to decrease the purchase of the SSBs (Fig 1); the majority of those who reported being aware of the tax (53.1%) indicated that they thought it was not reducing purchases of SSBs. The percentage of respondents who thought that the SSB tax was reducing purchases of SSBs was significantly greater among individuals aware of the SSB tax (12.1%) than among those not aware of the SSB tax (8.2%).

In the analyses stratified by socio-demographic characteristics (Table 2), chi-squared tests of independence revealed statistical significant differences between awareness and opinion about the SSB tax and sex (χ^2^ = 30.366, p = 0.019), SES (χ^2^ = 306.593, *p* < .001), area (χ^2^ = 87.617, *p* < .001), region (χ^2^ = 113.116, p = 0.002), and age (χ^2^ = 178.097, *p* < .001). The percentages of respondents who were aware of the SSB tax were significantly higher among people of high SES (74.4%), and living in Mexico City (76.6%) and in urban areas (67.5%).

**Table 2:** Awareness of the SSB tax and opinion about whether it was reducing purchases of SSBs, stratified by socio-demographic characteristics*.

				*Aware* of the SSB tax				*NOT aware of the SSB tax*			
		*Total*		*Believe* the SSBs tax is reducing purchases of SSBs		*DO NOT believe* the SSB tax is reducing purchases of SSBs		*Believe* the SSBs tax is reducing purchases of SSBs		*DO NOT believe* the SSB tax is reducing purchases of SSBs	
		Unweighted *n*	Weighted (in MM) *n*	*%*	(*95% CI*)	*%*	(*95% CI*)	*%*	(*95% CI*)	*%*	(*95% CI*)
National		6,321	6.9	12.1	(10.7–13.7)	53.1	(50.3–55.9)	8.2	(7.0–9.5)	26.6	(24.5–28.8)
Sex †											
	Male	2,054	27.6	12.0	(9.8–14.6)	55.9	(51.9–59.9)	6.6	(5.2–8.3)	25.5	(22.2–29.0)
	Female	4,267	29.8	12.2	(10.7–13.8)	50.5	(47.5–53.5)	9.7	(8.1–11.5)	27.7	(25.3–30.1)
SES †											
	Low ^a^	2,088	11.7	14.1	(12.2–16.2)	39.0	(35.2–42.9)	12.6	(10.1–15.6)	34.3	(30.8–37.9)
	Medium ^b^	2,175	16.8	12.7	(10.4–15.3)	45.3	(41.4–49.2)	9.0	(7.2–11.2)	33.0	(29.1–37.2)
	High ^c^	2,058	28.9	11.0	(8.8–13.5)	63.4 ^a,b^	(59.0–67.5)	5.9 ^a^	(4.3–8.0)	19.8 ^a,b^	(16.9–23.0)
Area †											
	Urban ^a^	3,213	44.6	11.4	(9.7–13.4)	56.1	(52.7–59.4)	7.1	(5.8–8.7)	25.3	(22.8–28.0)
	Rural	3,108	12.8	14.3	(12.7–16.1)	42.8 ^a^	(39.4–46.2)	11.9 ^a^	(9.9–14.2)	31.0	27.8–34.4)
Region *											
	North ^a^	1,372	12.2	11.6	(8.1–16.4)	50.7	(43.4–58.0)	9.2	(6.1–13.7)	28.5	(23.6–34.0)
	Center ^b^	2,083	18.7	12.9	(10.6–15.8)	53.4	(47.7–59.1)	6.9	(5.3–8.8)	26.8	(22.8–31.1)
	Mexico City ^c^	751	10.4	12.8	(9.6–16.8)	63.8 ^b,c^	(58.9–68.4)	5.8	(3.8–8.9)	17.6 ^a,b,c^	(13.5–22.6)
	South ^d^	2,115	16.1	11.0	(9.2–13.2)	47.7	(44.4–51.1)	10.5	(8.3–13.1)	30.8	(28.2–33.6)
Age †											
	20–29 ^a^	1,575	18.6	10.6	(8.6–13.0)	44.1 ^b,c,d^	(39.8–48.4)	10.4	(7.7–13.9)	34.9 ^b,c,d^	(31.0–39.1)
	30–39 ^b^	1,837	16.7	10.5	(8.2–13.3)	56.4	(50.6–62.0)	7.6	(5.9–9.7)	25.5	(21.5–30.0)
	40–49 ^c^	1,621	12.8	14.9	(11.7–18.9)	58.2	(54.0–62.3)	7.3	(5.5–9.7)	19.5	(15.9–23.6)
	50–59 ^d^	1,288	9.3	14.1	(11.5–17.1)	58.2	(53.5–62.7)	6.0	(4.6–7.8)	21.8	(18.2–25.8)
BMI											
	Normal weight	1,489	14.2	11.6	(9.0–14.7)	52.1	(47.3–56.9)	8.6	(6.6–11.1)	27.8	(23.5–32.6)
	Overweight	2,304	20.7	12.8	(10.7–15.1)	53.8	(49.6–57.8)	7.7	(6.9–9.7)	25.8	(22.7–29.2)
	Obesity	2,220	18.6	11.5	(9.4–13.9)	54.0	(48.7–59.2)	8.5	(6.1–11.7)	26.1	(22.5–30.0)

Notes.

SSB, sugar-sweetened beverages; MM, millions; SES, socio-economic status; BMI, body mass index.

Data are from the ENSANUT 2016: Mexican adults (20–59 years old).

* Values are percentages and 95% CIs. Percentages across a row sum up to 100.

† p < .05 based on χ^2^ test across categories. For each socio-demographic variable, different subscripts down a column (a, b, c, d) indicate statistically significant differences based on the 95% CIs (i.e. the CIs do not overlap).

### Factors associated with a self-reported decrease in SSB consumption

The final (parsimonious) logistic regression model only included statistically significant (or nearly significant) predictors and covariates. The final model was significant, *F*(10, 278) = 15.110, *p*< .001, and explained 9.4% (Nagelkerke pseudo-*R*^2^) of the variance in change (Table 3).

**Table 3:** Final, parsimonious, model for self-reporting a decrease in consumption of SSBs since the year the SSB tax was implemented, obtained by binary logistic regression* †.

		Consumption decreased *vs* consumption did not decrease since the year the SSB tax was implemented		
		*Odds Ratio*	*95% CI*	*P-*value
Awareness of the SSB tax				
	Aware	1.30	1.06, 1.59	.012
	Not aware	Reference		
Self-efficacy				< .001
	Very confident	1.68‡	1.15, 2.46	
	Confident	1.12	0.77, 1.64	
	Slightly confident	0.88	0.58, 1.35	
	Not confident	Reference		
Liking of SSBs				< .001
	Completely disagree	4.29‡	1.90, 9.70	
	Disagree	3.33‡	2.19, 5.01	
	Agree	1.68‡	1.23, 2.30	
	Completely agree	Reference		
Age		1.00	1.00, 1.02	.056
Diabetes				< .001
	Yes	1.77‡	1.33, 2.35	
	Yes—gestational	1.24	0.25, 6.10	
	No	Reference		
*R*^*2*^ Cox and Snell = 0.063				
*R*^*2*^ Nagelkerke = 0.094				

Notes.

SSB, sugar-sweetened beverages.

Data are from the ENSANUT 2016: Mexican adults (20–59 years old), *n* = 6,349.

* The full binary logistic regression model included seven variables (awareness of the tax, opinion about the effectiveness of the tax, self-efficacy, liking of SSBs, health-beliefs, and availability of free/low-cost potable water) and covariates (age, diabetes diagnosis, sex, socio-economic status, geographic region, and area). The reference category included adults who reported that their SSB consumption in the two years prior had decreased. The statistics presented are from the parsimonious model, which includes only statistically significant (or nearly significant) predictors and covariates.

† Values are Odd Ratios, 95% CIs, and *P*-values of variable effect in overall model based on Wald F test.

‡ Significant findings of subcategories based on the 95% CI (i.e. the CI does not include 1).

Among the six independent variables, three were statistically significant: awareness of the SSB tax, self-efficacy, and liking of SSBs. Respondents who were aware of the SSB tax were 30% more likely to report a decrease in consumption of SSBs in the two years prior. High self-efficacy and low liking of SSBs were also individually associated with a reported decrease in SSBs (*OR* = 1.68) and (*OR* = 4.29), respectively.

### Factors associated with current consumption of taxed SSBs

The final (parsimonious) model significantly predicted consumption of taxed SSBs, *F*(18, 262) = 32.51, *p* < .001, with *R*^2^ = 21.1% (Table 4).

**Table 4:** Final, parsimonious, model of factors associated with current consumption of taxed SSBs, obtained by multiple regression * † ‡.

		*β*	*% change in consumption of taxed SSBs* §	*SE* of the *β*	*P-value* (F test)	*P*-value (t tests)
Awareness of the SSB tax AND Area					.017	
	Urban (aware *vs* not aware)	-0.17	-15.72	0.08		.023
	Rural (aware *vs* not aware)	0.08	8.76	0.08		.250
Awareness of the SSB tax		Reference			.394	
Self-efficacy					< .001	
	Very confident	-0.76	-53.23	0.212		< .001
	Confident	-0.46	-36.87	0.11		< .001
	Slightly confident	-0.23	-20.55	0.13		.076
	Not confident	Reference				
Liking of SSBs					< .001	
	Completely disagree	-0.55	-42.31	0.28		.046
	Disagree	-0.73	-51.81	0.13		< .001
	Agree	-0.34	-28.82	0.10		.001
	Completely agree	Reference				
Urban-rural location		0.37	44.20	.090	< .001	
Sex (female *vs* male)		-0.60	-45.12	0.05	< .001	
Age		-0.02	-1.98	<0.01	< .001	
Region					< .001	
	North	0.42	50.20	0.11		< .001
	Center	0.20	22.14	0.08		.008
	Mexico City	-0.01	-1.00	0.09		.891
	South	Reference				
Diabetes					.005	
	Yes	-0.28	-24.42	0.09		.003
	Yes—gestational	1.04	182.92	0.96		.141
	No	Reference				
BMI					< .001	
	Obesity	0.31	36.34	0.09		< .000
	Overweight	0.10	10.52	0.09		.285
	Normal weight	Reference				
*R*^2^ *=* 0.211						

Notes.

SSBs, sugar-sweetened beverages; *β*, regression coefficient; *SE*, standard error; *R*^*2*^ proportion variance explained.

Data are from the ENSANUT 2016: Mexican adults (20–59 years old), *n* = 4,624

* The outcome variable, taxed SSB consumption (log g/person/day), was created combining the following variables: regular soda, industrialized nectars, industrialized fruit waters.

† The full multiple regression included seven variables (awareness of the tax, opinion about the effectiveness of the tax, self-efficacy, liking of SSBs, health-beliefs, and availability of free/low-cost potable water) and covariates (age, BMI, diabetes diagnosis, sex, socio-economic status, geographic region, and area). The statistics presented are from the final (parsimonious) model which includes only statistically significant variables.

‡ Values are *β* coefficients, % change in consumption of taxed SSBs (log g/person SSBs* (log g/person/day), SEs of the *β*s, *p-values* of each coefficient estimate in the Wald F test, and *p-values* of the t tests for each of the coefficients of the sub-categories within each factor. Lower *β*s indicate expectation of less SSB consumption/lower score on unfavorable behavior.

§ % change in consumption of taxed SSBs (log g/person/day) was calculated as: % *change in consumption of taxed SSBs* = (*e^β^* − 1) * 100.

Self-efficacy and liking of SSBs added significantly to the prediction (*p* < .001). Respondents who were very confident or confident in limiting their consumption of SSBs to <1 glass/week consumed less taxed SSBs (53.2% and 36.9%, respectively) than those who did not feel confident. Individuals who dislike SSBs consumed less (42.3%) than those who like them. A significant interaction between urban-rural location and awareness of the tax was found (*p* = .017), indicating that location of residence moderated the relationship between awareness of the SSB tax on consumption of taxed SSB beverages. In particular, only individuals living in urban areas a significant difference between those aware and not aware of the SSB tax was observed (a 15.7% decrease in taxed SSB consumption among those aware, compared to those who were not aware; *p* = .023).

## Discussion

### Awareness of the SSB tax and opinion about the impact of the SSB tax

Results show slightly more than sixty-five percent (65.2%) of adults reported being aware of the SSB tax. And while there are no equivalent country-level data, a study conducted about a year after the passing of an excise tax on SSBs in Berkley, California found a similar figure—68% of people interviewed knew that the tax had been on their city’s ballot [25]. It is possible that some respondents may have indicated they recalled the tax because it was the socially desirable response, but it’s worth noting that the tax was passed in the midst of very visible and controversial campaigns from proponents and opponents of the fiscal measure [17, 49]. According to Donaldson [17], the media campaign put forth by health advocates “generated over 1,000 media articles in the five-month period leading up to the vote on the tax… reaching the public as well as key decision-makers”. According to a Pan American Health Organization report [49] “the entire industry involved presented a united front against the tax, with very significant activism in the media—television, radio, press and advertising campaigns”.

In the current study, the largest percentage of respondents aware of the SSB tax was again found among people living in Mexico City and in urban areas, and of high SES. This finding is congruent with our hypothesis and can be explained by the fact that Mexico City was the stage of most of the advocacy and opposition campaigns, and that people of high SES living in urban areas might have had increased exposure and attentiveness to all the health messaging (print media, television, radio debates, etc.) about SSBs that went with the tax.

At national level, only 20.3% of respondents (combining those aware and unaware of the tax) thought that the fiscal measure was helping to decrease the purchase of SSBs. This finding may be explained by several potential factors. First, respondents reporting that the SSB tax was not reducing their purchases of SSBs could have made their judgment based on negative reports, articles and/or debates about the impact of the SSB tax. Second, they could have based their response on judgments of their own behavior and/or that of their peers. Third, in the past few decades, consumption of SSBs became deeply rooted in Mexican’ dietary habits [50], thus, in spite of an average 7.6% decrease in purchases of SSBs over the first two years [20], the perception might be that SSBs are still ubiquitous. Further qualitative/mixed methods studies are warranted to explore the reasons why most Mexican adults think the SSB tax is not working.

### Factors associated with a self-reported decrease in SSB consumption and with current consumption of taxed SSBs

Results of the binary logistic regression analysis showed that factors associated with a self-reported decrease in SSB consumption in the 2 years prior are: awareness of the SSB tax, high self-efficacy, and not liking of SSBs. Results of the multiple regression analysis showed that factors significantly associated with current consumption of taxed SSBs (log g/person/day) are: self-efficacy, liking of SSBs; and the interaction between awareness of the SSB tax and urban-rural area. In none of the models were opinion about the impact of the tax, health beliefs, and drinkable water availability significant.

Individuals *aware of the SSB tax* were 23% more likely to report a decrease in SSB consumption that those who were not aware. In addition, those aware of the tax, and living in urban areas, consumed 16.6% less taxed SSBs than people not aware. These findings suggest that the SSB tax and the publicity that surrounded it may have had a “signaling effect” thereby making people more conscious about their beverage choices. Our findings agree with the results of two prior studies that examined the impact of taxes on unhealthy food [24, 25]. An impact assessment of a tax on unhealthy non-staple food products passed in Hungary, found that 22–38% of consumers (depending on food categories) had reduced their intake of taxed products due to an increased health consciousness [24]. In the US city of Berkeley, a stronger than expected reduction in consumption of SSBs after the passing of SSB tax was partly attributed to the pro-tax media campaign, which, according to the study authors, may have shifted social norms and increased overall health consciousness [25]. Nevertheless, causality between awareness of a SSB tax and consumption of SSBs cannot be established, as people with a priori favorable attitudes and behaviors might have been more likely to pay attention to campaigns and debates.

*Opinion about whether the SSB tax was reducing SSB purchases* was not a significant predictor of reported change in SSB consumption since the year the tax was implemented. Two plausible explanations for this finding are that even if there has been a considerable decrease in purchases of SSBs—7.6% on average over the first 2 years since the introduction of the tax [20]—the change in participants’ purchases (in number of units or volume) of taxed beverages may have been small and not clearly noticeable to them, or perhaps there has not been a large enough critical mass who have changed their behaviors so as to have precipitated a change in a social norm that is so deeply entrenched [51, 52]. In this regard, it should be noted that when the ENSANUT 2016 was conducted the tax had already lost a small percent of its value because of inflation—the tax was adjusted in January 2018 after it rose 10 percent inflation from the time of implementation.

*Liking of SSBs* was a strong significant predictor of a self-reported decrease in SSB consumption in the 2 years prior to the survey, and also of current consumption of taxed SSBs. Studies have found that taste is one of the primary drivers of SSBs consumption [40, 53]. This is not surprising given that humans are genetically predisposed to prefer sweet taste [54]. However, preference is also learned [54]. In Mexico, there is a high exposure to sweetened beverages, starting from infanthood [55, 56]. Therefore, interventions and programs should focus on reducing children’s repeated exposure to SSBs to prevent heightened SSB preferences early in life from developing. In addition, it is not certain whether a liking for sweet taste can be reduced, thus efforts should be aimed at improving individuals’ self-efficacy and self-regulation skills.

*SSB-health related beliefs* were not associated with either a self-reported change in consumption of SSBs or current consumption of taxed SSBs. There are two plausible reasons for the absence of significance. One is that while beliefs about health outcomes or risks of behavior are a precondition for change, they are not enough on their own, and self-efficacy is needed to overcome impediments or barriers to adopting and maintaining healthy behaviors [57]. A second reason could be that there was little variation in the health beliefs data: 83% of all survey respondents believed that drinking SSBs is associated with the four diseases/conditions they were asked about (See S2 Table).

*Self-efficacy* was also a strong, significant predictor in both regression models. This suggests that people may have felt that they had the confidence to limit their SSB consumption if they wanted to, and, that if they have decreased SSB consumption, it might have been because they had a high sense of self-control. To explore whether people who reported being self-efficacious were those who did not drink SSBs, we conducted further analyses eliminating individuals with low consumption of SSBs (≤50 g/person/day), and the results remained significant (data not shown). Self-efficacy has been shown to be significantly associated with SSB consumption in other studies conducted in adults. For example, a study with US parents (*n* = 66) of adolescents found that among parents perceived behavior control was a significant predictor of SSB consumption and was significantly correlated with intention to decrease SSB consumption [38]. In another study with adults (*n* = 199), Zoellner et al. [39] found that perceived behavioral control was significantly associated with SSB consumption. While drinking (or stopping drinking) SSBs is not a complex behavior in itself, the innate preference for sweet taste and the important sociocultural aspects of SSB drinking in Mexico makes drinking less SSBs a challenging behavior change. Given that the awareness about the detrimental health consequences of drinking SSBs in this population is high (83% of respondents believed that SSBs contribute to obesity, HPB, diabetes, and dental caries), future public health efforts to increase knowledge and enhance motivation should be complemented by programs to assist individuals develop self-efficacy and self-regulation skills [57]. Nutrition education is therefore called for to help individuals develop self-efficacy and self-regulation skills, as well as to help them recognize their susceptibility to disease based on their current SSB consumption.

Overall, the regression models explained a modest amount of variation in the data (9% in self-reported change of SSB consumption, and 21% in current consumption of taxed SSBs). Nevertheless, the existing quantitative psychosocial models of dietary behavior change report a predictive validity less than 30% [58] suggesting that the results of this study are in line with the literature and indicates that the processes underlying food choice are complex and influenced by many factors.

### Limitations and strengths

There are several limitations to this study that should be considered when interpreting its results. First, the data from the POCAA-Q survey are self-reported, and thus could be subject to recall and social desirability response biases. Second, the associations are cross-sectional and do not permit assessment of causality or ascertaining the direction of the association. Third, the study did not use a pre-post design; thus, it was unable to assess change in measures before and after the SSB tax. Fourth, a post-only comparison of outcomes between those aware and not aware of the SSB tax does not fully take into account individuals with a priori favorable attitudes and behaviors who might have been more likely to pay more attention to the campaign. Fifth, there were other public health interventions aimed at decreasing consumption of SSBs that were implemented around the same time as the SSB tax. Lastly, the preference and self-efficacy constructs were assessed with only one item each; according to some researchers this may not adequately define a construct that is stable enough to use in future studies [59, 60].

Despite these limitations, the study has several strengths. Foremost, it provides the first analysis of awareness of the Mexican SSB tax and opinion about its effectiveness in reducing purchases of SSBs in addition to its relationships with a self-reported change in SSB consumption and with current consumption among Mexican adults. It is also the first to assess the association of self-efficacy, taste preference, and health beliefs with SSB consumption in Mexico on a national scale. Findings are generalizable nationally because the ENSANUT 2016 survey uses a probabilistic representative sample.

### Conclusions

Our findings suggest that accompanying SSB taxes with highly visible educational/informational campaigns may contribute to amplifying their effect by further reducing consumption of SSBs. Similarly, studies of tobacco control initiatives have suggested that while tobacco taxation and smoke-free places were two of the key elements of tobacco control strategies [61], part of the success could also be attributed to a shift in social norms and attitudes that emanated from policy initiatives and health education campaigns [62]. Further research is needed to understand the signaling effect of taxes and the influence of the publicity of taxes on SSB consumption but the aforementioned research lends support to suggested educational campaigns. Researchers in countries that are about to pass SSB taxes could more thoroughly examine this phenomenon by employing pre/post designs. The use of mixed-method approaches for the study of this complex phenomenon—beverage choice in the context of SSB taxes—is advised.

In addition, we found that SSB health-related beliefs were not significantly associated with either a self-reported decrease in SSB consumption after the implementation of the SSB tax, or to current consumption of taxed SSBs. Self-efficacy, on the other hand, and liking of SSBs, were significantly associated. In this context, where a majority of the Mexican adult population likes SSBs, drinks them frequently, and possesses knowledge about the detrimental consequences of SSBs consumption, public health and nutrition education campaigns designed to increase knowledge and enhance motivation should be complemented by programs to assist individuals develop self-efficacy and self-regulation skills.

## Supporting information

S1 Table Operational definitions and rationale of variables of choice from the perception of obesity, physical activity and questionnaire (POCAA-Q). ENSANUT 2016.
(DOCX)

S2 Table Percentages and (unadjusted) mean consumption of taxed SSBs in people self-reporting a decrease (or no decrease) in consumption of SSBs in the 2 years prior, by various characteristics. (DOCX)
