# Supplementary material for: Does the Mexican sugar-sweetened beverage tax have a signaling effect? ENSANUT 2016
Source: PLoS One. 2018 Aug 22;13(8):e0199337. doi: 10.1371/journal.pone.0199337 (PMC6104929; doi:10.1371/journal.pone.0199337)
Supplement: S1 Table — ENSANUT 2016. (DOCX) [file pone.0199337.s001.docx]

**S1 Table. Operational definitions and rationale of variables of choice from the Perception of obesity, physical activity and questionnaire (POCAA-Q). ENSANUT 2016.**

| **Variable/construct** | | **Survey Question(s)/Statement & Valid Response Options** | **Descriptions & Rationale** |
| --- | --- | --- | --- |
| Self-reported change in consumption of SSBs in the 2 years prior to the survey * | | “¿En los dos últimos años, usted considera que su consumo de bebidas azucaradas disminuyó, se mantuvo, o incrementó?” (In the past two years, do you think that your consumption of sugary beverage‡ has decreased, stayed the same, or increased?)  Valid response options: decreased, stayed the same, increased. | There was no pre-test data available to compare current with past consumption of SSBs. Self-reported change in consumption provides a proxy of change, although it does not provide information on change in quantity and frequency. Other studies had inquired about changes in past consumption of SSBs using the same question (1). |
| **SSB tax** | |  |  |
|  | Awareness of the SSB tax † | “¿Sabía usted que desde el año de 2014 hay un impuesto sobre el precio de las bebidas azucaradas (refrescos, jugos y aguas endulzadas)?” (Did you know that since 2014 there is a tax on industrialized sugary drinks such as sodas, juices and flavored water?)  Valid response options: yes, no. | Key variable in this study to assess whether people knew that a tax on SSBs was implemented in 2014. |
|  | Opinion about the potential of the SSB to reduce purchases of SSBs * | “¿Considera que esta acción está ayudando a disminuir la compra de bebidas azucaradas?” (Do you think that this [tax] is helping to reduce purchases of sugary beverages?)  Valid response options: yes, no. | Key variable in this study to assess people’s opinion about the potential of the SSB tax to reduce SSB purchases. It is considered a proxy of social norms. |
| **Psychosocial determinants** * | |  |  |
|  | Health beliefs | “Por favor, dígame si considera que el consumir bebidas azucaradas favorece el desarrollo de: (1) presión alta, (2) obesidad, (3) diabetes (azúcar alta en sangre), (4) caries dental” (Please, tell me if you think that SSB consumption contributes to the development of: (1) high blood pressure, (2) obesity, (3) diabetes (high blood sugar), (4) dental caries)  Valid response options: yes, no. | The theoretical construct “health belief" was operationalized as the *negative physical* *outcome expectations* of a dietary habit (2). The evidence regarding the role of health beliefs in SSB consumption is adult is scant; some studies have found significant associations (3). This association has never been studied in Mexican adults at national scale in Mexico. The health beliefs chosen (SSBs contribute to high blood pressure, obesity, and dental caries) are based on evidence of the link between a high SSB consumption and those conditions (4-10). |
|  | Self-efficacy (confidence to drink < 1 glass of SSBs per week) | “¿Qué tan capaz se siente de limitar su consumo a uno o menos vasos a la semana de bebidas azucaradas como refrescos, jugos y aguas endulzadas?” (How confident do you feel about drinking one or less glasses of sugary drinks (such as sodas, juices, nectars, and sweetened water) a week?”)  Valid response options: very confident, confident, somewhat confident, not confident. | Self-efficacy is the confidence to carry out the intended behavior successfully or overcome barriers to engaging in the behavior. It is a psychosocial construct from social cognitive theory (2); in the theory of planned behavior/reasoned action approach (11) it is known as perceived behavioral control.  Some studies have found that self-efficacy is significantly associated with SSB consumption and decrease (12, 13). However, literature in this regard is scant. No studies have examined this association in Mexican adults at a national scale. |
|  | Liking of SSBs | “¿Está de acuerdo con la afirmación: `El sabor de las bebidas azucaradas me gusta´” (Do you agree with this statement “You like the taste of sugary drinks”)  Valid response options: completely agree, agree, disagree, completely disagree. | Liking (also known as taste preference) is one of the strongest determinants of SSB consumption (14, 15). |
| **Environmental determinant** * | | |  |
|  | Availability of free/low-cost potable water in community | “¿Está de acuerdo con la afirmación: `Puedo beber agua potable de forma gratuita o a bajo costo en mi comunidad´” (Do you agree with the statement “I can drink potable water in my community at no cost or for free”?  Valid response options: completely agree, agree, disagree, completely disagree. | Potable drinking water is the recommended substitute for SSBs, but access to free/low-cost sources of potable water are not available across the board in Mexico. Some studies have found that consumption of industrialized SSBs is higher in places where access to free potable drinking water is limited and/or where people mistrust the safety of the water supply (16). |

*Notes.*

* “Don’t know” and no responses were converted to missing.

† “Don’t know” responses were converted to “no”, and no responses were converted to missing.

‡ In the questionnaire, the term “sugary drinks” was used instead of the technical term sugar-sweetened beverages. A description of the different categories of beverages included in the term “sugary drinks” was provided the first time it was used and two additional times throughout the questionnaire.

In the survey, the questions were presented in the following order: (1) self-efficacy, (2) perception of change in consumption of SSBs in the two years prior, (3) liking of SSBs, (4) availability of free/low-cost potable water in community, (5) health beliefs, (6) awareness of the tax, and (7) opinion about the effectiveness of the tax.

**References**

1. Boles M, Adams A, Gredler A, Manhas S. Ability of a mass media campaign to influence knowledge, attitudes, and behaviors about sugary drinks and obesity. Preventive medicine. 2014;67 Suppl 1:S40-5.

2. Bandura A. Self-efficacy: toward a unifying theory of behavioral change. Psychol Rev. 1977;84(2):191-215.

3. Park S, Onufrak S, Sherry B, Blanck HM. The relationship between health-related knowledge and sugar-sweetened beverage intake among US adults. J Acad Nutr Diet. 2014;114(7):1059-66.

4. Hu FB. Resolved: there is sufficient scientific evidence that decreasing sugar-sweetened beverage consumption will reduce the prevalence of obesity and obesity-related diseases. Obes Rev. 2013;14(8):606-19.

5. Greenwood DC, Threapleton DE, Evans CE, Cleghorn CL, Nykjaer C, Woodhead C, et al. Association between sugar-sweetened and artificially sweetened soft drinks and type 2 diabetes: systematic review and dose-response meta-analysis of prospective studies. Br J Nutr. 2014;112(5):725-34.

6. Imamura F, O'Connor L, Ye Z, Mursu J, Hayashino Y, Bhupathiraju SN, et al. Consumption of sugar sweetened beverages, artificially sweetened beverages, and fruit juice and incidence of type 2 diabetes: systematic review, meta-analysis, and estimation of population attributable fraction. BMJ (Clinical research ed). 2015;351:h3576.

7. Malik VS, Popkin BM, Bray GA, Despres JP, Willett WC, Hu FB. Sugar-sweetened beverages and risk of metabolic syndrome and type 2 diabetes: a meta-analysis. Diabetes Care. 2010;33(11):2477-83.

8. Wang M, Yu M, Fang L, Hu RY. Association between sugar-sweetened beverages and type 2 diabetes: A meta-analysis. J Diabetes Investig. 2015;6(3):360-6.

9. Huang C, Huang J, Tian Y, Yang X, Gu D. Sugar sweetened beverages consumption and risk of coronary heart disease: a meta-analysis of prospective studies. Atherosclerosis. 2014;234(1):11-6.

10. Moynihan PJ, Kelly SA. Effect on caries of restricting sugars intake: systematic review to inform WHO guidelines. J Dent Res. 2014;93(1):8-18.

11. Fishbein M, Ajzen I. Predicting and Changing Behavior: The Reasoned Action Approach. New York (USA): Taylor & Francis Group; 2010.

12. Riebl SK, MacDougal C, Hill C, Estabrooks PA, Dunsmore JC, Savla J, et al. Beverage Choices of Adolescents and Their Parents Using the Theory of Planned Behavior: A Mixed Methods Analysis. J Acad Nutr Diet. 2016;116(2):226-39 e1.

13. Zoellner J, Estabrooks PA, Davy BM, Chen YC, You W. Exploring the theory of planned behavior to explain sugar-sweetened beverage consumption. J Nutr Educ Behav. 2012;44(2):172-7.

14. Zoellner J, Krzeski E, Harden S, Cook E, Allen K, Estabrooks PA. Qualitative application of the theory of planned behavior to understand beverage consumption behaviors among adults. J Acad Nutr Diet. 2012;112(11):1774-84.

15. Block JP, Gillman MW, Linakis SK, Goldman RE. "If it tastes good, I'm drinking it": qualitative study of beverage consumption among college students. J Adolesc Health. 2013;52(6):702-6.

16. Onufrak SJ, Park S, Sharkey JR, Sherry B. The relationship of perceptions of tap water safety with intake of sugar-sweetened beverages and plain water among US adults. Public Health Nutr. 2014;17(1):179-85.
