# Supplementary material for: Does the Mexican sugar-sweetened beverage tax have a signaling effect? ENSANUT 2016
Source: PLoS One. 2018 Aug 22;13(8):e0199337. doi: 10.1371/journal.pone.0199337 (PMC6104929; doi:10.1371/journal.pone.0199337)
Supplement: S2 Table — (DOCX) [file pone.0199337.s002.docx]

**S2 Table. Percentages and (unadjusted) mean consumption of taxed SSBs in people self-reporting a decrease (or no decrease) in consumption of SSBs in the 2 years prior, by various characteristics.***

|  | | **Total (*n =* 6550)** | | | **People who decreased in SSBs** | | **People who did not decrease in SSBs** † | | **Total (*n =* 4867)** | | **People who decreased in SSBs** | **People who did not decrease in SSBs*** |
| --- | --- | --- | --- | --- | --- | --- | --- | --- | --- | --- | --- | --- |
|  | | **Unweighted *n*** | **Weighted (in MM) *n*** | ***%*** | ***%*** | **(*95% CI*)** | ***%*** | **(*95% CI*)** | **Unweighted *n*** | **Weighted (in MM) *n*** | ***Mean* taxed SSB (g/day)± *SEM*** | ***Mean* taxed SSB (g/day)± *SEM*** |
| Awareness of the SSB tax | |  |  |  |  |  |  |  |  |  |  |  |
|  | Aware | 3,944 | 38.0 | 64.2 | 44.8 | (40.9-48.8) | 55.2 | (51.2-59.1) | 2,925 | 25.6 | 310.7± 25.0 | 509.8± 29.1 |
|  | Not aware | 2,617 | 21.0 | 35.8 | 37.9 | (34.7-41.2) | 62.1 | (58.8-65.3) | 1,903 | 16.1 | 318.4± 36.9 | 596.1± 44.6 |
| Opinion about the effect of the SSB tax | |  |  |  |  |  |  |  |  |  |  |  |
|  | Yes | 1,586 | 11.6 | 20.3 | 42.4 | (38.5-46.4) | 57.6 | (53.6-61.5) | 1,142 | 8.7 | 345.4± 52.6 | 462.3± 48.8 |
|  | No | 4,709 | 45.6 | 79.7 | 42.2 | (38.8-45.7) | 57.8 | (54.3-61.2) | 3,522 | 34.8 | 314.5± 22.9 | 565.2± 28.7 |
| Health Beliefs (scale) | |  |  |  |  |  |  |  |  |  |  |  |
|  | 0 – “No” to all 4 Qs | 279 | 2.2 | 4.0 | 34.5 | (47.8-29.4) | 65.5 | (76.8-70.6) | 188 | 1.6 | 390.8± 152.2 | 514.1± 137.7 |
|  | 1 – “Yes” to 1 Q | 111 | 1.0 | 1.8 | 29.4 | (45.4-46.2) | 70.6 | (82.7-53.8) | 83 | 0.8 | 479.6± 205.1 | 615.9± 198.4 |
|  | 2 – “Yes” to 2 Qs | 158 | 1.2 | 2.2 | 46.2 | (58.7-41.2) | 53.8 | (65.7-58.8) | 100 | 0.7 | 237.4± 62.1 | 793.6± 165.7 |
|  | 3 – “Yes” to 3 Qs | 621 | 5.2 | 9.3 | 41.2 | (48.6-43.5) | 58.8 | (65.9-56.5) | 448 | 3.7 | 316.8± 50.5 | 533.8± 71.1 |
|  | 4 – “Yes” to all 4 Qs | 5,002 | 46.5 | 82.7 | 43.5 | (46.7-42.7) | 56.5 | (59.6-57.3) | 3,708 | 35.8 | 314.5± 23.1 | 544.7± 28.1 |
| Self-efficacy | |  |  |  |  |  |  |  |  |  |  |  |
|  | Very confident | 1,822 | 18.9 | 32.0 | 51.7 | (45.8-57.5) | 48.3 | (42.5-54.2) | 1,181 | 12.7 | 271.2± 36.2 | 451.7± 57.0 |
|  | Confident | 2,694 | 22.4 | 38.2 | 40.3 | (36.9-43.8) | 59.7 | (56.2-63.1) | 2,034 | 17.9 | 306.8± 24.9 | 500.3± 30.5 |
|  | Slightly confident | 1,530 | 13.2 | 22.5 | 34.7 | (30.8-38.9) | 65.3 | (61.1-69.2) | 1,237 | 10.5 | 369.2± 56.3 | 569.3± 33.7 |
|  | Not confident | 459 | 4.3 | 7.3 | 35.0 | (27.5-43.4) | 65.0 | (56.6-72.5) | 351 | 3.6 | 427.1± 61.7 | 896.7± 146.9 |
| Liking of SSBs | |  |  |  |  |  |  |  |  |  |  |  |
|  | Strongly agree | 4,323 | 9.2 | 15.8 | 28.7 | (22.6-35.8) | 71.3 | (64.2-77.4) | 630 | 7.4 | 423.4± 73.4 | 842.8± 91.1 |
|  | Agree | 1,291 | 39.0 | 65.8 | 40.5 | (37.6-43.5) | 59.5 | (56.5-62.4) | 3,321 | 30.6 | 347.2± 25.5 | 484.8± 22.3 |
|  | Disagree | 158 | 9.4 | 16.1 | 59.4 | (22.6-35.8) | 40.6 | (34.5-47.0) | 797 | 5.9 | 168.8± 23.2 | 344.7± 43.6 |
|  | Strongly disagree | 6,549 | 1.4 | 2.3 | 67.3 | (22.6-35.8) | 32.7 | (64.2-77.4) | 73 | 0.9 | 157.0± 35.0 | 226.3± 58.6 |
| Availability of free/low-cost potable water | |  |  |  |  |  |  |  |  |  |  |  |
|  | Strongly agree | 951 | 9.2 | 2.6 | 39.8 | (33.2-46.9) | 60.2 | (53.1-66.8) | 715 | 7.3 | 294.7± 35.9 | 659.2± 101.5 |
|  | Agree | 4,584 | 40.3 | 13.3 | 42.7 | (39.7-45.8) | 57.3 | (54.2-60.3) | 3,371 | 30.7 | 344.7± 26.3 | 531.0± 27.8 |
|  | Disagree | 857 | 7.8 | 68.4 | 44.5 | (35.1-54.2) | 55.5 | (45.8-64.9) | 627 | 5.7 | 177.1± 31.2 | 468.5± 56.2 |
|  | Strongly disagree | 142 | 1.5 | 15.7 | 36.5 | (27.4-46.7) | 63.5 | (53.3-72.6) | 96 | 1.0 | 301.4± 105.3 | 464.5± 99.1 |

*Notes*.

SSBs, sugar-sweetened beverages; MM, millions; SEM, standard error of the mean; Q, question; Qs, questions.

Data are from the ENSANUT 2016: Mexican adults (20-59 years old).

* Values are percentages (they sum up to 100 across rows), and unadjusted means and SEMs. The total samples sizes of the percentages and the means are different because the means are calculated with data from the SFFQ data file which has fewer cases than the POCAA-Q data file.

† The “did not decrease” category was calculated combining the “decrease” and “stayed the same” categories of the variable perception of change in consumption of SSBs.
